# Resolutions of Respect to Dr. Theodore F. Chupein

**Published:** 1901-08

**Authors:** 


### RESOLUTIONS OF RESPECT TO DR. THEODORE F. CHUPEIN.

Whereas, With regret we note the death of Dr. Theodore F. Chupein, an honorary member of the Academy, who departed this life March 23, 1901; and

Whereas, Dr. Chupein has ever been an active and progressive member of the dental profession, thoroughly devoted to its interests, and who by his earnestness, his interest in society work, and his ability as a writer, as evidenced by his many contributions to dental literature, did well merit the honorary membership conferred upon him when the Academy was organized; therefore

*Resolved,* As the sense of this society, that by the death of Dr. Chupein the Academy has lost a distinguished member and the profession a faithful follower. That as a man Dr. Chupein was affable, kindly, and unselfish, and as a practitioner skilful, conscientious, and enthusiastic in all that tended to professional advancement.

*Resolved,* That these resolutions be recorded upon our minutes and published with the proceedings of the Academy.

(Signed) WM. H. TRUEMAN.
S. H. GUILFORD.
C. N. PEIRCE.

